# Extract from a Review of Dr. Gross’s Work on the Urinary Organs

**Published:** 1852-08

**Authors:** 


### THE MEDICAL EXAMINER.

### PHILADELPHIA, AUGUST, 1852.

We transfer to our pages, with great pleasure, the following extract from a review of Dr. Gross's work on the Urinary Organs, in the July No. of the British and Foreign Medico-Chirurgical Review, as evidence of a kindlier feeling on the part of foreign journals towards American authors. The reviewer has been speaking of an English work on a kindred subject, by Dr. Coulson, in connection with which he says:

"It has remained for an American writer to wipe away this reproach; and so completely has the task been fulfilled, that we venture to predict for Dr. Gross's treatise a place in the literature of surgery, worthy to rank with the best works of the present age. Not merely is the matter good, but the getting up of the volume is most creditable to transatlantic enterprise; the paper and print would do credit to a first rate London establishment; and the numerous wood cuts which illustrate it demonstrate that America is making rapid advances in this department of art. We have, indeed, unfeigned pleasure in congratulating all concerned in this publication, on the result of their labors; and experience a feeling something like what might animate a long expectant husbandman, who, oftentimes disappointed by the produce of a favorite field, is at last agreeably surprised by a stately crop, which may bear comparison with any of its former rivals. The grounds of our high appreciation of the work will be obvious as we proceed; and we doubt not that the present facilities for obtaining American books will induce many of our readers to verify our recommendation by their own perusal of it."

